# The association of cerebral embolic protection during transcatheter aortic valve replacement with periprocedural neurological outcomes

**DOI:** 10.3389/fcvm.2026.1881371

**Published:** 2026-08-17

**Authors:** Funda Baysal, Kira Osipenko, Severin Laengle, Irene Steiner, Amila Kahrovic, Paul Werner, Philipp Bartko, Daniel Zimpfer, Martin Andreas, Iuliana Coti

**Affiliations:** 1 Department of Cardiac and Thoracic Aortic Surgery, Medical University of Vienna, Vienna, Austria; 2 Center for Medical Data Science, Institute of Medical Statistics, Medical University of Vienna, Vienna, Austria; 3 Department of Internal Medicine II, Medical University of Vienna, Vienna, Austria; 4 Department of Cardiac Surgery, Medical University of Graz, Graz, Austria

**Keywords:** cerebral embolic protection, delirium, ischemic stroke, transcatheter aortic valve implantation, transient ischemic attack

## Abstract

**Introduction:**

Cerebral Embolic Protection Devices (CEPs) have been designed to minimize the risk of periprocedural stroke. The clinical significance of these devices, however, is still under debate. We aimed to compare periprocedural neurological outcomes and mortality in patients undergoing transfemoral transcatheter aortic valve replacement (TF-TAVR) with vs. without CEP.

**Methods:**

A single-center retrospective analysis of 1,101 patients undergoing transfemoral TAVR from August 2017 to May 2025 was performed. CEPs were used routinely at our institution whenever anatomically feasible beginning with October 2019. The primary outcome was defined as the incidence of ischemic stroke occurring within 3 days postoperatively. Secondary endpoints included transient ischemic attack (TIA) and delirium within 3 days and short-term all-cause mortality.

**Results:**

Overall, 809 underwent TF-TAVR with CEP, while 292 were treated without. The primary endpoint of clinical ischemic stroke occurred less frequently in the CEP group (1.4% vs. 4.1%), and the group difference revealed a significant result in a univariable Cox regression analysis (*p* = 0.007). No clinically relevant differences were observed in the incidence of TIA (0.5% vs. 0.7%, *p* = 0.71) and postprocedural delirium (1.6% vs. 2.4%, *p* = 0.39). The 30-day mortality rate was numerically higher in the control group, but the difference did not reach statistical significance (1.9% vs. 3.9%, *p* = 0.06).

**Conclusion:**

The use of neuroprotection during TAVR was associated with a lower observed hazard of early periprocedural ischemic stroke in this unadjusted retrospective cohort.

## Graphical Abstract

**Figure:**
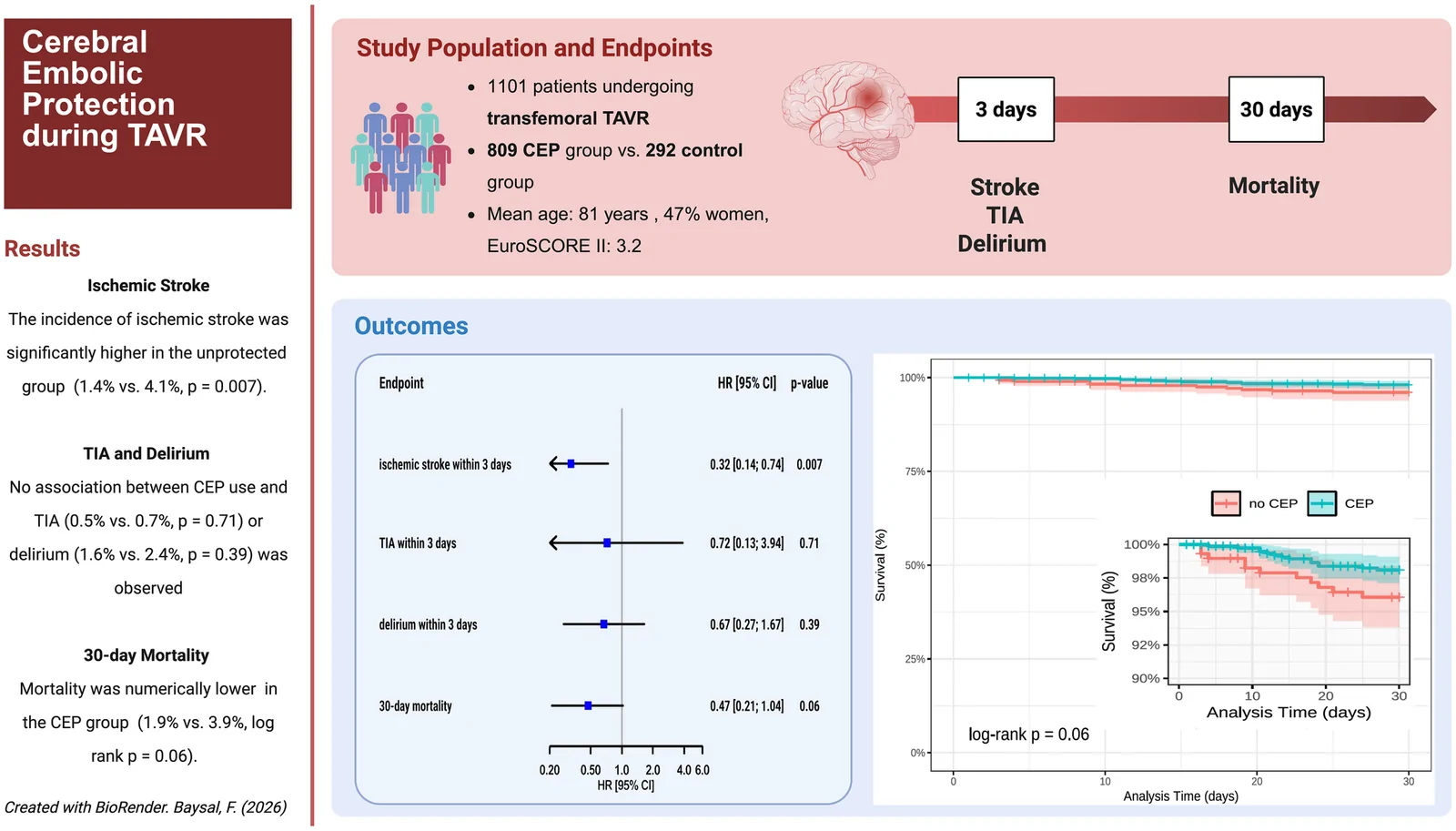
Impact of Cerebral Embolic Protection Devices on Periprocedural Neurological Outcomes and 30-day Mortality. Created with BioRender. Baysal, F. (2026) https://BioRender.com/vk853yo licensed under CC BY 4.0.

## 1 Introduction

Stroke remains one of the most feared complications after TAVR. Recent technological advances have therefore increasingly focused on strategies to prevent thromboembolic events. Understanding the mechanism of cerebrovascular complications following TAVR is essential for developing effective preventive strategies (1). Early postprocedural strokes are predominantly ischemic and might be driven by procedural factors such as catheter or valve manipulation, rapid pacing, balloon dilatation and mechanical interaction with the aorta—which can dislodge debris into the cerebral circulation (2).

CEPs were developed to reduce the risk of periprocedural stroke by capturing embolic material. Imaging based randomized clinical trials (3–7) have demonstrated the feasibility and safety of routine CEP use during TAVR, while mitigating new lesion formation on magnetic resonance imaging (MRI).

Despite these encouraging findings, substantial controversy persists regarding the overall effectiveness of CEPs. While imaging data and several observational cohort studies support routine use, the two most recent large scaled randomized clinical trials have failed to show a significant reduction in clinical apparent stroke. In the PROTECTED TAVR trial (8), which randomized 3,000 patients, the Sentinel device (Boston Scientific) did not significantly reduce stroke within 72 h. Consistent with this, the BHF PROTECT-TAVI (9), enrolling approximately 7,000 patients, also reported no difference in overall stroke events and disabling stroke between the CEP and control group.

Given that post-TAVR stroke increases in-hospital mortality nearly threefold (10), and that TAVR is increasingly performed in younger patient populations, further evidence is needed to clarify the clinical impact of neuroprotection devices and to identify the patient subgroups most likely to benefit from CEP.

## 2 Materials and methods

### 2.1 Study population

The present study is a retrospective data analysis including patients who underwent TAVR from August 2017 to May 2025 at the Department of Cardiac and Thoracic Aortic Surgery at the Medical University of Vienna. For this analysis we excluded patients treated with TAVR via a non-transfemoral approach, and those with an unsuccessful procedure defined by failure of valve delivery or intraprocedural switch to surgical aortic valve replacement (SAVR).

### 2.2 Data source

Data on baseline characteristics, operative details, and adverse events have been collected retrospectively within the framework of the ongoing VICTORY-II register (Ethics Number: 1680/2020), which was approved by the ethics committee of the Medical University of Vienna.

At hospital admission patients gave a written informed consent and undergone a baseline clinical fragility assessment. After valve implantation, patients are prospectively followed up at discharge, 30 days, 6 months, 1 year and then yearly up to 10 years.

### 2.3 Operative technique

The indication for TAVR was determined by an interdisciplinary heart team in accordance with current guidelines. Transfemoral TAVR was performed percutaneously—most commonly via the right femoral artery under conscious sedation and local anaesthesia. Both self-expandable and balloon-expandable devices were used. Pre- and post-dilatation was applied when necessary.

Since October 2019, cerebral embolic protection has been routinely implemented at our center. Aortic arch anatomy and branch vessels morphology were assessed by computed tomography (CT), and neuroprotection was omitted in patients with unsuitable anatomy such as severe stenosis and tortuosity, soft plaques or when vascular access was insufficient.

Two different neuroprotection devices were used. The Sentinel device (Boston Scientific, Marlborough, MA), inserted via the right radial artery which consists of two filter units with 140-µm pores, that are positioned in the brachiocephalic trunk and left common carotid artery. In contrast, the TriGuard 3™ device (Keystone Heart Ltd., Caesarea, IL, USA) is a deflection device inserted via the femoral artery and positioned in the aortic arch to cover all three large branches.

### 2.4 Definitions and outcomes

All outcome variables were defined according to the Valve Academic Research Consortium-3 (VARC 3) criteria. Neurologic events followed the Neurologic Academic Research Consortium (NeuroARC) classification and were categorized as:

- Overt CNS injury (NeuroARC Type 1)
- Covert CNS injury (NeuroARC Type 2)
- Neurological dysfunction without CNS injury (NeuroARC Type 3)

The primary endpoint was clinical ischemic stroke within 3 days after TAVR defined as an acute focal or multifocal neurological deficit consistent with a vascular territory in the brain, spinal cord, or retina (NeuroARC Type1a or 1aH). Secondary endpoints included transient ischemic attack (Type 3a/3aH) and delirium (Type 3b), representing neurological dysfunction without CNS injury according to VARC 3, assessed within 3 days.

All patients with new neurological symptoms underwent CT or MRI to confirm central nervous system (CNS) injury and determine etiology. As no routine postoperative neuroimaging was performed, asymptomatic patients were not further evaluated, and covert CNS injuries (Type 2) or so-called silent strokes were not included. Delirium was retrospectively ascertained from documented clinical diagnoses in medical records without systematic CAM-ICU screening.

Short-term mortality refers to all-cause death after valve implantation and includes in-hospital mortality and mortality at 30-days.

### 2.5 Statistical analysis

Continuous variables have been reported using mean and standard deviation (SD) if approximately normally distributed or median and interquartile range (first quartile Q1; third quartile Q3) if otherwise. For group comparisons, Welch t-tests or Wilcoxon rank sum tests were performed, respectively. Categorical variables have been reported using absolute frequencies and percentages. Differences have been assessed using Fisher's exact tests.

To evaluate the association of neuroprotection with the primary outcome, a univariable Cox-regression model with neuroprotection as independent variable was calculated. The dependent variable was the number of days from valve implantation (day 0) until the occurrence of ischemic stroke within an observation period of 3 days. Patients without event were censored at the earliest of re-surgery (3 patients needed re-surgery within 2 days post-TAVR), death or day 3 post-surgery, respectively. In patients with an event occurring on the day of valve implantation, the time frame from surgery until event was set to 0.5 days. An estimate for the hazard ratio with 95% confidence intervals (CI), as well as the *p*-value (H0: hazard ratio is equal to 1) was reported. Graphical visualization was done by Kaplan Meier plots, whereby a log-rank test was calculated to compare the survival curves. The incidence of ischemic stroke by day 3 post-surgery was calculated as 1 minus Kaplan–Meier estimates, additionally 95% confidence intervals are reported. The secondary endpoints overall neurological events within 3 days, TIA within 3 days, and delirium within 3 days were analyzed analogously. Furthermore, the associations of the following baseline variables with the primary endpoint and with overall neurological events within 3 days, respectively, were analyzed by univariable Cox regression models: age, sex, BMI, Euro Score II (log-transformed), STS Score (log-transformed), Porcelain aorta, Agatston Score, diabetes mellitus, insulin dependent diabetes mellitus, peripheral vascular disease, cerebrovascular disease, history of stroke/ TIA, atrial fibrillation/ flutter, coronary artery disease, previous myocardial infarction, CABG, PCI, congestive heart failure, LVEF < 50%, chronic kidney disease, creatinine (log-transformed), dialysis, bicuspid aortic valve, oral anticoagulation with NOAC, oral anticoagulation with VKA, single antiplatelet therapy, valve-in-valve procedure, small valve size (≤23 mm), use of balloon-expandable valve, balloon dilatation before valve implant, balloon dilatation after valve implant, and postoperative new onset atrial fibrillation. In case of complete separation, a Cox regression model with Firth correction was applied (R-function logistf, R-package logistf version 1.26.1).

To evaluate the association of 30-day mortality and neuroprotection, a univariable Cox-regression model was calculated with neuroprotection as independent factor. The dependent variable was the number of days from valve implantation (day 0) until death within the following 30 days. Patients without event were censored at the earliest of re-surgery (4 patients had a re-surgery at two or four days post-TAVR, respectively), last observation or day 30, respectively. Additionally, univariable Cox-regression models were calculated to analyze the following baseline variables: age, sex, BMI, diabetes mellitus, COPD, coronary artery disease, atrial fibrillation/flutter, chronic kidney disease, dialysis, PVL end of OP, dialysis immediately after TAVR, Euro Score II (log-transformed), STS (log-transformed), creatinine (log-transformed). To analyze the association of neurological outcomes, all neurological events within 3 days and ischemic stroke within 3 days with 30-day mortality, univariable Cox regression models were calculated with the aforementioned variables included as time-dependent covariables in the model. In-hospital mortality was compared between patients with vs. without CEP as described above. In-hospital mortality was calculated as the number of days from valve implantation (day 0) until death. Patients without event were censored at the earlier of discharge (internal or external) or re-surgery (5 patients). Median length of stay (range) was calculated as days from surgery until death, re-surgery or discharge, respectively.

The results of the univariable Cox regression models of the primary and secondary endpoints have been depicted using forest plots.

As we started using CEP in October 2019, we additionally conducted a subset analysis of patients who underwent TAVR between 2019 and 2025. The results are available in the Supplementary Data.

Because eligibility for CEP was determined by anatomical suitability, the positivity assumption required for propensity score methods was not met. Therefore, no propensity score methods were applied. Given the limited number of events, particularly for the primary endpoint, univariable rather than multivariable Cox regression models were performed to avoid overfitting and unstable effect estimates.

Missing data were handled using complete-case analysis. No data imputation has been performed. A *p*-value of ≤0.05 was considered as significant in all statistical tests. Due to the exploratory character of the study, no adjustment for multiple testing was conducted. The interpretation of the *p*-values is exploratory. Statistical analyses were performed using R statistical software version 4.5.1.

## 3 Results

### 3.1 Study cohort

A total of 1,107 patients underwent TF-TAVR at our department between August 2017 and May 2025. Six patients with unsuccessful valve deployment were excluded. In five patients, annulus rupture occurred during valve implantation resulting in conversion to SAVR, while in one patient the valve implantation became technically challenging, so the procedure had to be aborted.

Baseline characteristics are summarized in Table 1. Overall, age and sex were equally distributed with a mean age of 81 years in both groups and 52.7% male patients. The median EuroScore II and Society of Thoracic Surgeons score (STS) for the overall cohort were 3.2 (IQR 1.9–5.5) and 8.8 (IQR 7.2–11.1), respectively. Baseline operative risk did not differ substantially between both groups, overall indicating an intermediate to high surgical risk cohort.

**Table 1:** Baseline characteristics.

Variables	Total	Cerebral embolic protection (CEP)	Non-cerebral embolic protection (non-CEP)	*p*-value
	*N* = 1,101	*N* = 809	*N* = 292	
Age (years), mean	80.86 ± 6.69	80.86 ± 6.39	80.85 ± 7.46	0.988
Sex: male	580 (52.7%)	433 (53.5%)	147 (50.3%)	0.374
BMI (kg/m^2^), mean number of missing values	27.05 ± 4.87 5	27.0 ± 4.84 4	27.18 ± 4.95 1	0.583
BSA (m^2^), mean number of missing values	1.87 ± 0.21 5	1.87 ± 0.21 4	1.86 ± 0.21 1	0.900
EuroScore II (%), median number of missing values	3.2 (1.9–5.5) 75	2.9 (1.9–5.3) 70	3.7 (1.9–6.1) 5	0.053
STS score, median	8.8 (7.2–11.1)	8.8 (7.2–11.0)	8.9 (7.5–11.2)	0.126
NYHA Class III-IV Number of missing values	621 (62.0%) 99	442 (60.7%) 81	179 (65.3%) 18	0.189
Previous cardiac surgery	121 (11.0%)	92 (11.4%)	29 (9.9%)	0.585
Previous coronary revascularization CABG PCI	384 (34.9%) 87 (7.9%) 337 (30.6%)	288 (35.6%) 66 (8.2%) 255 (31.5%)	96 (32.9%) 21 (7.2%) 82 (28.1%)	0.431 0.704 0.300
Previous balloon valvuloplasty	6 (0.5%)	3 (0.4%)	3 (1.0%)	0.194
Porcelain aorta	28 (2.5%)	19 (2.3%)	9 (3.1%)	0.517
Diabetes mellitus Insulin-dependent	337 (30.6%) 91 (8.3%)	257 (31.8%) 65 (8.0%)	80 (27.4%) 26 (8.9%)	0.182 0.622
Hyperlipidemia	859 (78.0%)	634 (78.4%)	225 (77.1%)	0.680
Hypertension	981 (89.1%)	728 (90.0%)	253 (86.6%)	0.125
COPD	173 (15.7%)	119 (14.7%)	54 (18.5%)	0.134
Home oxygen use	18 (1.6%)	13 (1.6%)	5 (1.7%)	1.000
Coronary artery disease	485 (44.1%)	359 (44.4%)	126 (43.2%)	0.731
Previous myocardial infarction	133 (12.1%)	106 (13.1%)	27 (9.2%)	0.094
Peripheral vascular disease	180 (16.3%)	123 (15.2%)	57 (19.5%)	0.097
Cerebrovascular disease	267 (24.3%)	189 (23.4%)	78 (26.7%)	0.265
History of stroke or transient ischemic attacks	145 (13.2%)	107 (13.2%)	38 (13.0%)	1.000
Atrial fibrillation/flutter	403 (36.6%)	284 (35.1%)	119 (40.8%)	0.089
Congestive heart failure	321 (29.2%)	252 (31.1%)	69 (23.6%)	**0** **.** **016**
LVEF < 50% Number of missing values	244 (24.7%) 114	178 (25.0%) 98	66 (23.9%) 16	0.743
Chronic kidney disease	359 (32.6%)	276 (34.1%)	83 (28.4%)	0.081
Dialysis	27 (2.5%)	15 (1.9%)	12 (4.1%)	**0** **.** **045**
Creatinine (mg/dL), median Number of missing values	1.1 (0.9–1.4) 7	1.1 (0.9–1.4) 3	1.1 (0.9–1.5) 4	0.231
Native bicuspid aortic valve	21 (1.9%)	18 (2.2%)	3 (1.0%)	0.316
Oral anticoagulation with NOAC Number of missing values	362 (33.8%) 31	281 (36.0%) 28	81 (28.0%) 3	**0** **.** **016**
Oral anticoagulation with VKA Number of missing values	34 (3.2%) 31	20 (2.6%) 28	14 (4.8%) 3	0.076
Single antiplatelet therapy Number of missing values	421 (39.3%) 31	308 (39.4%) 28	113 (39.1%) 3	0.944
Dual antiplatelet therapy Number of missing values	157 (14.7%) 31	116 (14.9%) 28	41 (14.2%) 3	0.846

Boldface indicates statistical significance (*p* ≤ 0.05).

Metric variables are summarized as mean ± standard deviation if approximately normally distributed. The *p*-value is derived from a Welch t-test. For skewed data, median (first quartile–third quartile) and the *p*-value derived from a Wilcoxon rank sum test are reported. Categorical variables are summarized as absolute frequencies and percentages, *p*-values correspond to Fisher's exact tests. BMI, body mass index; BSA, body surface area; STS, Society of Thoracic Surgeons; NYHA, New York Heart Association; CABG, coronary artery bypass grafting; PCI, percutaneous coronary intervention; COPD, chronic obstructive pulmonary disease; LVEF, left ventricular ejection fraction; NOAC, non-vitamin K antagonist oral anticoagulants; VKA, vitamin K antagonist oral anticoagulants.

The proportion of patients with end-stage renal disease on dialysis was higher in the unprotected cohort (4.1% vs. 1.9%, *p* = 0.045), while more patients had congestive heart failure (31.1% vs. 23.6%, *p* = 0.016) in the CEP group.

Postoperative antithrombotic management differed significantly between both groups. Compared with the CEP group, the non-CEP group more frequently received oral anticoagulation with vitamin K antagonists (VKA) (7.7% vs. 2.0%, *p* < 0.001) and dual antiplatelet therapy (DAPT) (41.3% vs. 22.9%, *p* < 0.001), whereas single antiplatelet therapy (SAPT) was more common in the CEP group (45.0% vs. 30.8%, *p* < 0.001).

**Figure 1:**
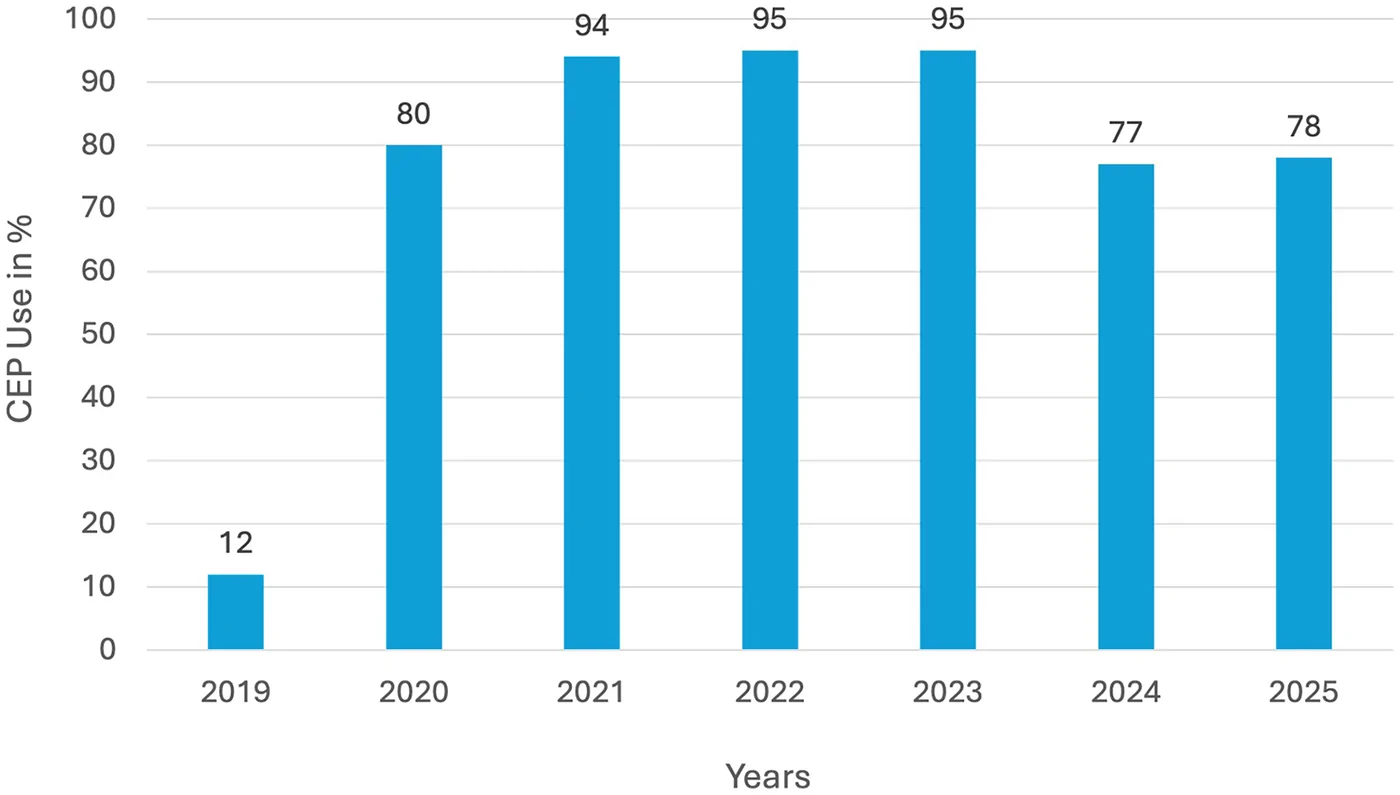
Neuroprotection use over time. CEP use began in October 2019 reaching a plateau of 95% CEP TAVR from 2021 to 2023. CEP, cerebral embolic protection.

Overall, 809 (73.48%) patients underwent TF-TAVR with CEP, while 292 (26.52%) were performed without protection. Sentinel device accounted for the vast majority of CEP cases (92.6%), whereas the TriGuard system was used in 7.4%. As illustrated in Figure 1, CEP adoption expanded rapidly after its introduction in October 2019, stabilizing at approximately 95% use between 2021 and 2023. With increasing operator experience and improved identification of anatomical contraindications, CEP use declined modestly in 2024.

Procedural characteristics were similar between groups (Table 2). More than 96% of procedures were not emergent. Valve-in-valve TAVR was performed in 70 patients (6.4%) and pre- and post-implantation balloon dilatation rates were 41.1% and 21.0%, respectively. In 25.1% of patients, a balloon-expandable valve was implanted. Intraoperative paravalvular leakage (≥moderate) after valve implantation occurred in 16 patients (1.7%).

**Table 2:** Procedural characteristics.

Variables	Total	Cerebral embolic protection (CEP)	Non-cerebral embolic protection (non-CEP)	*p*-value
	*N* = 1,101	*N* = 809	*N* = 292	
Urgency Elective Urgent Emergent	1,060 (96.3%) 34 (3.1%) 7 (0.6%)	780 (96.4%) 25 (3.1%) 4 (0.5%)	280 (95.9%) 9 (3.1%) 3 (1.0%)	0.587
Operative time (min), mean	57.49 ± 25.58	57.27 ± 26.26	58.11 ± 23.64	0.630
Valve in valve	70 (6.4%)	49 (6.1%)	21 (7.2%)	0.487
Combined procedures	17 (1.5%)	11 (1.4%)	6 (2.1%)	0.412
Small valve size (≤23 mm) number of missing values	149 (13.6%) 2	108 (13.4%) 2	41 (14.0%) 0	0.766
Balloon-expandable valve	276 (25.1%)	197 (24.4%)	79 (27.1%)	0.386
Pre-dilatation	453 (41.1%)	325 (40.2%)	128 (43.8%)	0.298
Post-dilatation	231 (21.0%)	165 (20.4%)	66 (22.6%)	0.451
PVL ≥ moderate at the end of the procedure number of missing values	15 (1.4%) 1	10 (1.2%) 0	5 (1.7%) 1	0.559
Oral anticoagulation with NOAC (postoperative) number of missing values	376 (34.5%) 11	290 (36.1%) 5	86 (30.1%) 6	0.070
Oral anticoagulation with VKA (postoperative) number of missing values	38 (3.5%) 11	16 (2.0%) 5	22 (7.7%) 6	<**0.001**
Single antiplatelet therapy (postoperative) number of missing values	450 (41.3%) 11	362 (45.0%) 5	88 (30.8%) 6	<**0.001**
Dual antiplatelet therapy (postoperative) number of missing values	302 (27.7%) 11	184 (22.9%) 5	118 (41.3%) 6	<**0.001**

Boldface indicates statistical significance (*p* ≤ 0.05).

For categorical variables, absolute frequencies and percentages are reported. The *p*-values have been calculated with Fisher's exact tests. Metric variables are summarized as mean ± standard deviation, and the *p*-value is derived from a Welch *t*-test. PVL, paravalvular leak, NOAC, non-vitamin K antagonist oral anticoagulants; VKA, vitamin K antagonist oral anticoagulants.

### 3.2 Primary outcome—ischemic stroke

Most neurological events occurred within the first 24 h. In total, 49 neurological events within 3 days have been recorded. One patient in the CEP cohort developed both postoperative delirium and ischemic stroke. No hemorrhagic stroke occurred during the 3 days observation period.

**Figure 2:**
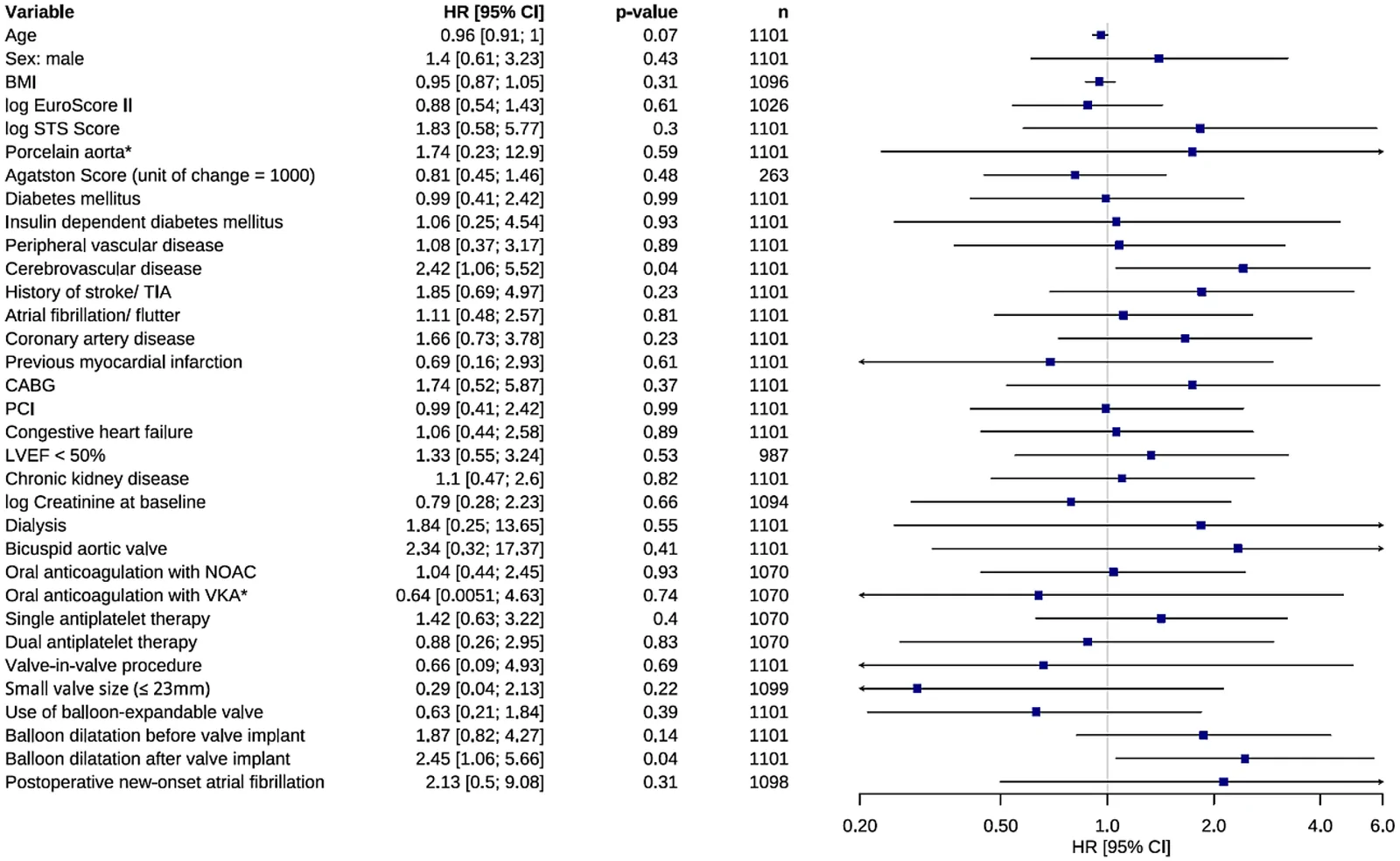
Univariable Cox regression analysis for ischemic stroke within 3 days. Forest plot depicting hazard ratios for ischemic stroke occurring within 3 days after TAVR for prespecified baseline variables. Squares indicate HR and horizontal lines represent 95% CI. *P* values of the univariable Cox regression models are displayed. (*) indicates that a Cox regression model with Firth correction was applied. BMI, body mass index; STS, Society of Thoracic Surgeons; TIA, transient ischemic attack; CABG, coronary artery bypass grafting; PCI, percutaneous coronary intervention; LVEF, left ventricular ejection fraction; NOAC, non-vitamin K antagonist oral anticoagulants; VKA, vitamin K antagonist oral anticoagulants.

Overall, 23 patients presented with ischemic stroke within 3 days after TAVR. The incidence was markedly higher in the unprotected group, compared with those treated with CEP (1 − Kaplan–Meier estimate: 4.1% [95% CI: 1.8%; 6.4%] vs. 1.4% [0.6%; 2.2%]). In univariable Cox regression analysis, in the cohort treated with CEP a significantly reduced risk of early ischemic stroke [HR 0.32, 95% CI: (0.14–0.74), *p* = 0.007] was observed compared to the unprotected group. Balloon post-dilatation [HR 2.45, 95% CI: (1.06–5.66), *p* = 0.04] and cerebrovascular disease [HR 2.42, 95% CI: (1.06–5.52), *p* = 0.04] were univariably associated with ischemic stroke in the Cox regression analyses (Figure 2).

Since neuroprotection devices were introduced in 2019 at our department, a time-dependent selection bias could not be ruled out. To address this, we conducted a subset analysis of patients undergoing TAVR between 2019 and 2025. The results were concordant with those of the entire study cohort and confirmed a significant negative association of ischemic stroke (*p* = 0.005) with CEP use in the univariable Cox regression analysis (Supplementary Table 1).

Furthermore, we performed a subgroup analysis whether the incidence of ischemic stroke differed between the two CEP devices. Ischemic stroke occurred in 9 of 749 patients (1.2%) treated with the Sentinel device and 2 out of 60 patients treated with TriGuard device. Although the incidence was lower in the Sentinel cohort, (1 − Kaplan–Meier estimate: 1.2% [95% CI: 0.4%; 2%] vs. 3.3% [95% CI: 0; 7.8%], *n* = 809), the estimates are based on limited number of events.

### 3.3 Secondary outcomes

#### 3.3.1 TIA and delirium

No significant differences were observed in the incidence of TIA (1 − Kaplan–Meier estimate [95% CI]: 0.5% [0%; 1%] vs. 0.7% [0%; 1.6%], Cox regression: *p* = 0.71) and delirium (1 − Kaplan–Meier estimate [95% CI]:1.6% [0.7%; 2.5%] vs. 2.4% [0.6%; 4.1%], *p* = 0.39) between groups. Outcome rates and corresponding hazard ratios and *p* values are summarized in Table 3.

**Table 3:** Primary and secondary outcomes.

Variables	Cerebral embolic protection (CEP)	Non-cerebral embolic protection (non-CEP)	HR [95% CI]	*p*-value
	*N* = 809	*N* = 292		
Ischemic stroke within 3 days	11 (1.4%)	12 (4.1%)	0.32 [0.14; 0.74]	0.007
Transient ischemic attack within 3 days	4 (0.5%)	2 (0.7%)	0.72 [0.13; 3.94]	0.71
Delirium within 3 days	13 (1.6%)	7 (2.4%)	0.67 [0.27; 1.67]	0.39
30-day mortality	14 (1.9%)	11 (3.9%)	0.47 [0.21; 1.04]	0.06

Counts represent the observed number of patients with each event. The percentage of events (%) are derived from the Kaplan–Meier curve (1 − Kaplan–Meier estimate). Hazard ratios (HRs) and *p*-values were derived from univariable Cox regression analyses.

#### 3.3.2 Mortality

There was no statistically significant difference in in-hospital mortality between both groups [HR 0.68, 95% CI: (0.3; 1.51), *p* = 0.34]. In total, 24 patients died during hospital stay. The median length of hospital stay was 4 days (range: 1–94 days) in the CEP group and 2 days in the control group (range: 1–107 days).

**Figure 3:**
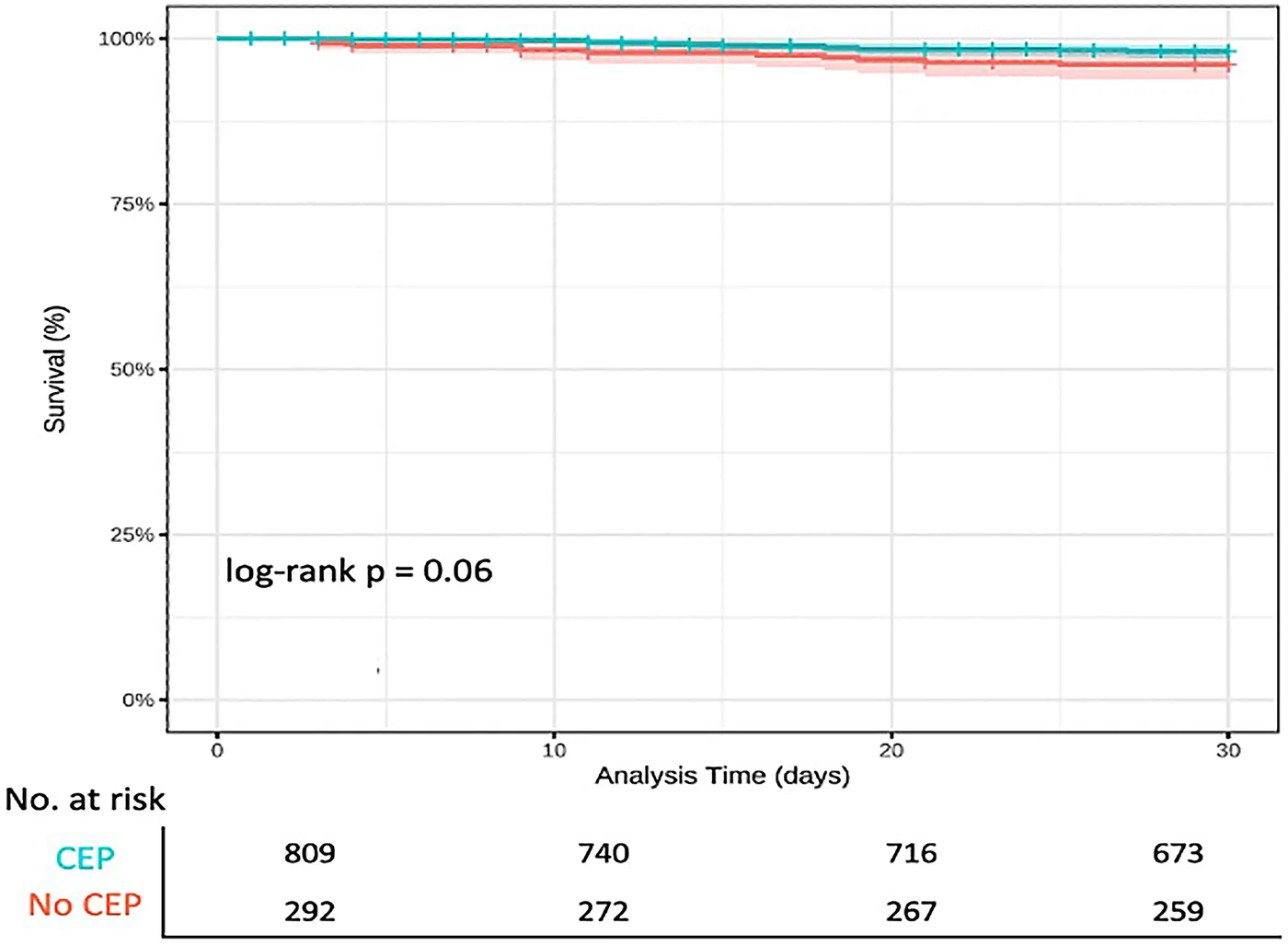
Kaplan–meier survival curve for 30-Day mortality. Left: total sample, Right: blue: transfemoral TAVR with CEP, Red: transfemoral TAVR without CEP. Survival at 30 days follow up does not significantly differ between patients undergoing TAVR with or without neuroprotection devices—log-rank *p* = 0.06.

The 30-day mortality rate was higher in patients undergoing TAVR without neuroprotection (1.9% [0.9%; 2.9%] with CEP vs. 3.9% [1.6%; 6.2% without CEP]), however, no significant univariable association between the use of neuroprotection and 30-day mortality could be identified [HR 0.47, 95% CI:(0.21–1.04), *p* = 0.06]. The Kaplan–Meier survival curves for both groups have been depicted in Figure 3.

**Figure 4:**
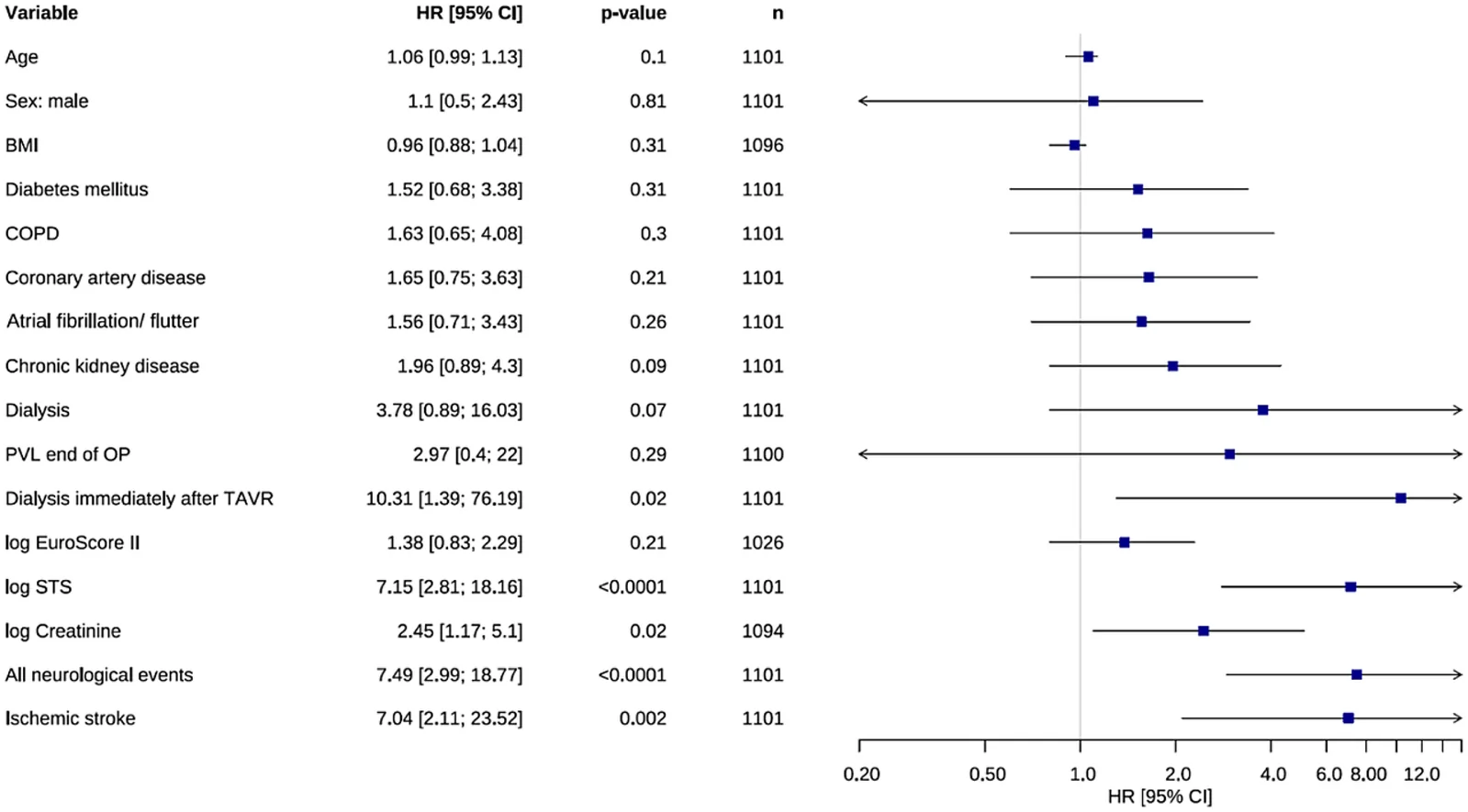
Univariable Cox regression analysis for 30-day All-cause mortality. Forest plot depicting hazard ratios for all-cause mortality within 30 days after TAVR for prespecified baseline variables. Squares indicate HR and horizontal lines represent 95% CI. *P* values of the univariable Cox regression models are displayed. (*) indicates univariable Cox-regression models with time-dependent covariable. BMI, body mass index; COPD, chronic obstructive pulmonary disease; PVL, paravalvular leak; STS, Society of Thoracic Surgeons.

STS score [HR 7.15, 95% CI: (2.81–18.16), *p* < 0.0001] and elevated baseline creatinine [HR 2.45, 95% CI: (1.17–5.1), *p* = 0.02] as well as new-onset renal replacement therapy after TAVR [HR 10.31, 95% CI: (1.39–76.19), *p* = 0.02] were univariably associated with 30-day mortality. Ischemic stroke within 3 days was strongly associated with increased 30-day mortality [HR 7.04, 95% CI: (2.11–23.52), *p* = 0.002] (Figure 4).

Furthermore, the Kaplan–Meier curves of patients treated between 2019 and 2025 revealed a significantly lower 30-day mortality rate in the CEP group (1.9% [0.9% 2.9%] vs. 4.7% [1%; 8.4%] Cox regression: HR 0.38, 95% CI: (0.14–0.98), (*p* = 0.05)) (Supplementary Figure 1).

#### 3.3.3 Technical success

After the introduction of CEP devices at our department, the decision to use CEP was primary guided by anatomical considerations. Contraindications for CEP use included significant stenosis of the left common carotid or brachiocephalic artery, dissection, aneurysm or ectasia of the aortic arch and its supra-aortic branches as well as inadequate access site.

Overall, 134 patients have been operated without neuroprotection between October 2019 and May 2025. In 39 patients, CEP implantation had initially been planned, however, arterial access was deemed insufficient during intraoperative assessment due to significant stenosis, inadequate flow, small vessel diameter or severe tortuosity or kinking. CEP implantation was unsuccessful in two cases due to difficulty advancing the device into the brachiocephalic artery caused by vessel kinking while device malfunction occurred in two additional cases.

## 4 Discussion

This study provides several key findings: 1) Ischemic stroke within 3 days after TAVR was significantly reduced in the patient group for whom cerebral embolic protection (CEP) was possible; with an estimated relative risk reduction of >65%. 2) Early ischemic stroke was univariably associated with a higher risk of 30-day mortality.

Although technological refinements and operator experience have reduced many procedural complications, periprocedural stroke risk remained relatively stable at 2%–5% over the last decade, varying by patient risk profile (11). Early trials such as PARTNER-1A (12) reported higher stroke rates in TAVR compared with SAVR (5.5% vs. 2.4%, *p* = 0.04). More recent data from PARTNER II and III trials (13, 14) have shown lower rates (0.6%–3%), yet overall stroke incidence has not declined despite expansion of TAVR into younger, lower-risk cohorts (15).

Stroke has a multifactorial etiology, embolization of valve-, vessel- or procedure related debris is considered the primary mechanism (16). Consistent with prior reports, neurological events in our cohort occurred predominantly within 3 days, with more than half on the procedural day, mirroring findings from Huded et al. (17).

### 4.1 CEP and neurological outcomes

Since the introduction of CEP devices in 2012, multiple imaging, histopathologic and clinical studies have been conducted to test the feasibility and safety of neuroprotection devices. In the early feasibility studies, filters captured debris in 80%–99% of cases, including thrombus, tissue, and foreign material (4, 18).

Imaging-dedicated randomized clinical trials (RCTs), such as CLEAN TAVI (3), MISTRAL-C (5), REFLECT II (6) and DEFLECT III (7) showed that CEP reduced the number and volume of new MRI lesions and may mitigate neurocognitive decline. With TAVR increasingly implanted in younger patients, the potential long-term consequences of silent brain injuries linked to neurocognitive decline, are of growing relevance (19, 20). In MISTRAL-C trial neurocognitive deterioration was 7 times more frequent in the unprotected cohort (4% vs. 27%; *p* = 0.017) (5), whereas DEFLECT III trial showed better memory performance, fewer neurological deficits and an increased cognitive recovery with use of TriGuard (7). Lesion volume, in particular, has emerged as predictive marker for stroke, with larger total lesion burden >500 mm^3^ correlating strongly with increased and disabling stroke risk (21).

Large RCTs evaluating clinical endpoints, PROTECTED TAVR (8) and BHF PROTECT-TAVI (9) provide more nuanced results. PROTECTED TAVR showed a non-significant 22% relative reduction in stroke (2.1% and 2.7%), whereas our study observed a larger difference (1.4% [95% CI: 0.6%; 2.2%] vs. 4.1% [95% CI: 1.8%; 6.4%]). A *post-hoc* US-only analysis of PROTECTED TAVR by Makkar et al. (22) demonstrated a significant stroke reduction with CEP, suggesting regional differences in case selection, procedural strategies or baseline risk may have influenced results. Importantly, the PROTECTED cohort had a substantially lower baseline risk (mean STS ≈ 3%) than our population (mean STS ≈ 10%), which may explain the higher stroke rates in unprotected patients in our study. Notably, the PROTECTED trial did demonstrate a reduction in disabling stroke in the CEP group (0.5% vs. 1.3%, *p* = 0.02). However, the larger BHF PROTECT-TAVI trial, enrolling >7,600 patients, found no difference in overall (2.1% vs. 2.2%) or disabling stroke (1.2% vs. 1.4%). A substudy from BHF PROTECT- TAVI by Kennedy et al. (23) also reported no cognitive benefit with CEP, though the absence of systematic neuroimaging limits conclusions, given the high rate of subclinical cognitive decline observed. Both RCTs were ultimately underpowered due to unexpectedly low events rates; contemporary estimates suggest that >22,000 patients would be needed to detect a significant reduction in stroke with CEP at current incidence levels (24).

In contrast with RCTs, observational studies consistently report benefit with CEP. Multiple propensity-matched analyses show reductions in early stroke, neurological events and composite endpoints. Seeger et al. (25) observed significantly lower 7-day mortality or stroke with CEP (2.1% vs. 6.8%) and stroke rates nearly identical to ours (1.4% vs. 4.6%).

Larger meta-analyses further support CEP effectiveness. Shahid reported significantly reduced stroke and disabling stroke with CEP (26), while Kheyrbek highlighted a clear difference between RCT and observational data, with benefit confined primarily to real-world cohorts (27). Another meta-analysis pooling CLEAN-TAVI, MISTRAL-C, and PROTECTED showed reduced overall and disabling stroke with a notably lower number needed to treat than reported in PROTECTED TAVR (24). Our findings align closely with these observational results, reinforcing the signal of reduced early stroke with CEP.

Importantly, we used two different CEP systems (when available on the market) to adapt to patient's anatomy. This was not possible in company sponsored RCTs and may explain part of the observed difference. While the Sentinel system was routinely used, patients with unavailable radial access or soft plaques in the truncus were protected by the TriGuard system. Thereby, we were able to improve the overall percentage of patients protected, and reduced device related adverse events. On the other hand, the inclusion of two different devices is a potential source of heterogeneity. The devices employ different mechanisms of embolic protection. While the Sentinel device is filter-based, the TriGuard device provides a deflector-based protection of all three supra-aortic vessels. The Kaplan–Meier estimated incidence of ischemic stroke within 3 days was higher with TriGuard compared to Sentinel. However, the subgroup analysis was limited by the very low number of events (11 events), resulting in wide confidence intervals.

### 4.2 CEP and mortality

Periprocedural stroke is a strong determinant of mortality. Prior analyses have shown a >3-fold increase in 30-day mortality after stroke (28) consistent with our finding which showed that ischemic stroke within 3 days was univariably associated with 30-day mortality [HR 7.04, 95% CI: (2.11–23.52), *p* = 0.002]. While some earlier observational studies reported similar in-hospital and 30-day mortality rates between both groups (2, 29), two recent meta-analyses suggest that CEP may improve both, neurological events and all-cause mortality (26, 27).

However, no mortality benefit was observed in either of the two RCT. We found no significant difference in 30-day mortality between both groups. In the exploratory analysis of the subset (2019–2025) there was a trend towards lower 30-day mortality with neuroprotection devices, which is more likely explained by residual confounding.

## 5 Conclusion

In our study, lower hazard of early periprocedural ischemic stroke was observed among patients considered suitable for CEP use during TAVR compared with those not suitable, reflecting two clinically distinct cohorts. Importantly, ischemic stroke emerged as a significant predictor of 30-day mortality following TAVR. The clinical use of CEP during TAVR appears to be declining following the publication of the two aforementioned large, randomized trials, However, we strongly support a more individualized approach to CEP use. Patients with bicuspid aortic valves, severe valvular calcification requiring pre-and post-dilatation and valve-in-valve procedures may benefit the most. Future studies should focus on identifying anatomical and procedural characteristics associated with the greatest treatment benefit.

## 6 Limitations

We conducted a single-center retrospective analysis with inherent limitations. A key limitation of our study is the potential for residual confounding regarding patient selection and treatment era. The use of CEP was determined by our cardiac surgery team considering anatomy and technical feasibility. Therefore, the positivity assumption required for propensity score methods was not met, rendering propensity score matching and inverse probability of treatment weighting inappropriate. Consequently, patients undergoing TAVR with CEP and those undergoing TAVR alone cannot be considered exchangeable, and present analyses should be interpreted as a comparison of two clinically distinct cohorts, precluding a causal inference regarding the effect of CEP.

Furthermore, due to the limited number of outcome events, particularly for the primary outcome, univariable rather than multivariable Cox regression models were performed to avoid overfitting. These univariable analyses describe unadjusted associations between group (CEP vs. TAVR alone) and outcomes but do not allow conclusions regarding the independent effect of CEP, and residual confounding by both measured and unmeasured factors cannot be excluded. Since we started using CEP in October 2019, earlier patients were operated without, introducing a potential era effect, when comparing both groups. Differences in operator experience, procedural techniques, device technology and peri-procedural antithrombotic strategy may have contributed to outcome differences between both groups. It should be noted that the subset analysis of patients undergoing TAVR between 2019 and 2025 partly addresses time-related confounding. However, residual confounding by indication remains, as non-CEP cases were those in which CEP was considered anatomically unsuitable predominantly due to unfavorable supra-aortic anatomy or vascular access. As these anatomical characteristics are associated with an increased risk of cerebrovascular events, they may have influenced the observed outcomes independently of CEP use.

Due to the observational nature of our study, residual confounding related to patient selection, baseline heterogeneity in comorbidities, differences in postoperative antithrombotic strategy, device heterogeneity and treatment era, cannot be fully excluded and may have influenced the outcomes, potentially leading to an overestimation of the observed treatment effect, which is why our findings should be interpreted as hypothesis generating and not as definitive evidence of causal CEP effect.

Moreover, the diagnosis of postoperative delirium is based on medical records only as no routine standardized screening has been performed. Therefore, delirium may have been under-ascertained and should not be interpreted with the same level of diagnostic certainty as imaging-confirmed outcomes.

Further, missing data were handled using complete-case analysis without data imputation, which may have introduced bias if the missing data were not completely at random.

## Data Availability

Data supporting the findings of this study will be provided by the corresponding author upon request. Requests to access these datasets should be directed to Iuliana Coti, iuliana.coti@meduniwien.ac.at.

## Authors note

Meeting presentation: The abstract was accepted as ePoster at London Valves Meeting 2025 (ID A78740FB).
