# Supplementary material for: The association of cerebral embolic protection during transcatheter aortic valve replacement with periprocedural neurological outcomes
Source: Front Cardiovasc Med. 2026 Aug 17;13:1881371. doi: 10.3389/fcvm.2026.1881371 (PMC13527623; doi:10.3389/fcvm.2026.1881371)
Supplement: Supplementary file 1 [file Datasheet1.docx]

## Supplemental Material

| **Supplementary Table 1: Primary and Secondary Outcomes in the 2019-2025 Subset** | | | | |
| --- | --- | --- | --- | --- |
| **Variables** | **Cerebral Embolic Protection** (CEP)  N = 809 | **No Cerebral Embolic Protection** (no CEP)  N = 134 | **HR [95 % CI]** | **p-value** |
| Ischemic stroke within 3 days | 11 (1.4 %) | 7 (5.2 %) | 0.25 [0.1;0.65] | **0.005** |
| Transient ischemic attack within 3 days | 4 (0.5 %) | 1 (0.7 %) | 0.66 [0.07;5.92] | 0.71 |
| Delirium within 3 days | 13 (1.6 %) | 1 (0.7 %) | 2.16 [0.28;16.51] | 0.46 |
| 30-day mortality | 14 (1.9 %) | 6 (4.7 %) | 0.38 [0.14;0.98] | 0.05 |
| Event counts represent the observed number of events. Percentages of events with 95% CI derived from the Kaplan Meier curve (1- Kaplan-Meier estimate) for the subset 2019-2025. CEPs were implemented as standard practice at our institution whenever feasible starting in October 2019. Overall, 943 patients underwent transfemoral TAVR, while 809 received a neuroprotection device and 134 have been operated without. | | | | |

**Supplementary Figure 1:** Kaplan-Meier Survival Curve of 30-Day Mortality in the 2019-2025 Subset

Legend Supplementary Figure 1: Blue = transfemoral TAVR with CEP, Red = transfemoral TAVR without CEP. The 30-day mortality in patients without neuroprotection was significantly higher compared to patients undergoing TAVR with neuroprotection devices (log-rank p = 0.04).
